# Multimodal AI for Biomarker and Etiological Assessment of Dementias

**DOI:** 10.1093/geroni/igaf122.1248

**Published:** 2025-12-31

**Authors:** Vijaya Kolachalama

**Affiliations:** Boston University, Boston, Massachusetts, United States

## Abstract

Dementia differential diagnosis and biomarker assessment pose significant challenges due to symptom overlap and limited access to advanced imaging like PET for detecting amyloid beta (Aβ) and tau (τ) in neurodegenerative conditions such as Alzheimer’s disease (AD). In this talk, I will highlight our lab’s recent work on multimodal AI frameworks that fuse diverse data to identify dementia etiologies, handle mixed dementias and incomplete cases, and estimate Aβ/τ PET status. This approach promises scalable screening for personalized dementia management, AD therapies, and clinical trials.

